# Egas: a collaborative and interactive document curation platform

**DOI:** 10.1093/database/bau048

**Published:** 2014-06-11

**Authors:** David Campos, Jóni Lourenço, Sérgio Matos, José Luís Oliveira

**Affiliations:** ^1^BMD Software, Lda., Rua Calouste Gulbenkian n. 1, 3810-074 Aveiro, Portugal and ^2^IEETA/DETI, Campus Universitário de Santiago, University of Aveiro, 3810-193 Aveiro, Portugal

## Abstract

With the overwhelming amount of biomedical textual information being produced, several manual curation efforts have been set up to extract and store concepts and their relationships into structured resources. As manual annotation is a demanding and expensive task, computerized solutions were developed to perform such tasks automatically. However, high-end information extraction techniques are still not widely used by biomedical research communities, mainly because of the lack of standards and limitations in usability. Interactive annotation tools intend to fill this gap, taking advantage of automatic techniques and existing knowledge bases to assist expert curators in their daily tasks. This article presents Egas, a web-based platform for biomedical text mining and assisted curation with highly usable interfaces for manual and automatic in-line annotation of concepts and relations. A comprehensive set of de facto standard knowledge bases are integrated and indexed to provide straightforward concept normalization features. Real-time collaboration and conversation functionalities allow discussing details of the annotation task as well as providing instant feedback of curator’s interactions. Egas also provides interfaces for on-demand management of the annotation task settings and guidelines, and supports standard formats and literature services to import and export documents. By taking advantage of Egas, we participated in the BioCreative IV interactive annotation task, targeting the assisted identification of protein–protein interactions described in PubMed abstracts related to neuropathological disorders. When evaluated by expert curators, it obtained positive scores in terms of usability, reliability and performance. These results, together with the provided innovative features, place Egas as a state-of-the-art solution for fast and accurate curation of information, facilitating the task of creating and updating knowledge bases and annotated resources.

**Database URL:** http://bioinformatics.ua.pt/egas

## Introduction

A growing amount of biomedical data is continuously being produced, resulting largely from the widespread application of high-throughput techniques, such as gene and protein analysis. This growth is accompanied by a corresponding increase of textual information, in the form of articles, books and technical reports. To capture scientific evidences from all these sources, several manual curation efforts have been set up to identify concepts (e.g. genes and proteins), associated information (e.g. gene function) and relations (e.g. protein–protein) and finally store the extracted information in structured resources. However, manual annotation of large quantities of data is a demanding and expensive task (1, 2), being difficult to keep these databases up-to-date. For instance, (3) argue that manually curating and fulfilling some genomic resources may take decades to be completed. These factors have naturally led to an increasing interest in the application of text mining (TM) systems to perform those tasks automatically. However, because of the complexity of the domain and the ambiguity of the associated scientific documents, the automatic extraction of biomedical information remains challenging, even if high-performance results have been reached in some particular tasks. For instance, in the CRAFT (4) corpus, Neji (5) achieved 95% of F-measure in the recognition of species names and 76% of F-measure identifying gene and protein names. On the other hand, relation mining solutions present considerably inferior results, a direct consequence of the inherent task complexity. For instance, in the recognition of protein–protein interactions (PPIs), the solution presented by (6) achieved F-measures results from 51 to 84% in distinct corpora. When considering drug–drug interactions (DDIs) mining, the best solution (7) achieved 66% of F-mesure in the DDIExtraction corpus (8). Overall, the most advanced solutions still produce many mistakes that must be taken into account when updating existing knowledge bases. Thus, one must carefully analyse the provided automatic information and correct the existing mistakes. In this perspective, various studies have shown that using automatic solutions to assist biocurators delivers improved curation times (9, 10). Nevertheless, such solutions are still not being widely used by biomedical research communities (11), which are the main target audience. This gap is related not only with the complexity and ambiguity of biocuration tasks, but also with the lack of standards and interaction between biocurators and developers. Moreover, (12) showed that usability of bioinformatics resources is fundamental to effectively support users in their daily research activities. Thus, it is important to develop interactive solutions that take advantage of automatic computational solutions and existing knowledge resources to assist expert curators in their daily tasks. To do so, the interface with the curator is an important aspect that needs to be considered for tool adoption. In the end, by taking advantage of such interactive solutions, biocurators can easily and more effectively keep current knowledge bases updated and generate annotated data to develop and evaluate automatic solutions.

Various research groups have developed solutions to assist biocurators, following different approaches, providing different features and targeting different tasks. Overall, two general tasks have been tackled: document triage and information annotation. Triage intends to retrieve and rank documents considering a specific goal. For instance, the BioCreative challenges organized a task (13, 14) to automatically classify documents as relevant for PPI curation. On the other hand, information annotation targets identifying information contained in documents. Many challenges were organized targeting the automatic extraction of concepts (15–17), relations (8, 13, 14) and events (18–20). Brat (21) is one of the most complete web-based solutions for information curation, supporting in-line annotation of documents. It provides concept normalization features, automatic services integration, search capabilities and documents comparison. However, annotation task configuration (e.g. target concepts and relations, normalization resources and automatic services) is considerably difficult and non-accessible for non-advanced users, and document representation is considerably slow when full-text documents are used. MyMiner (22) is another complete web-based solution for biocuration, which supports concept tagging and normalization of a predefined set of concepts using a restrict set of previously processed resources. It also supports document triage, automatic concept recognition and document comparison. However, because interacting with annotations is only possible through a table rather than directly with in-line annotations, users may not have a direct view of the corresponding text and context, which makes understanding the inherent information considerably more difficult.

Following a different approach, Argo (23) offers workflow design options with previously built and integrated components. Thus, users are able to create custom-processing pipelines for concept and relation annotation with manual correction, supporting multiple import and export formats. Even though such approach is powerful, creating such workflows may require advanced expertise and provides a high level of flexibility that may not be required for biocurators. Other solutions, such as BioQRator (http://www.bioqrator.org), CellFinder (http://141.20.31.85/cellfinder), PubTator (24), RLIMS-P (http://research.bioinformatics.udel.edu/rlimsp), tagtog (https://www.tagtog.net) and Ontogene (25) follow typical web-based solutions with less usable interactions and annotation representation, using tabular listings of concept and/or relation annotations with simple highlighting and sorting/scoring capabilities. Nonetheless, some of those solutions incorporate interesting features. For instance, BioQRator integrates document triage for PPIs, tagtog integrates active learning of concept names using annotated information and PubTator features a PubMed-like interface with many state-of-the-art automatic solutions already integrated for concept recognition and normalization. There are other solutions that do not apply classic web-based approaches. For instance, SciKnowMine (http://www.isi.edu/projects/sciknowmine/overview) is a desktop application for document triage that integrates active learning capabilities to obtain new models based on interactively annotated documents. On the other hand, MarkerRIF (http://bws.iis.sinica.edu.tw/MarkerRIF) is a web-browser extension that allows annotating concepts directly on documents from the Pubmed website, providing relevant sentences retrieval and supporting normalization of a restrict set of concepts.

Overall, in addition to the features of these tools, several desirable characteristics can be identified that should facilitate the wider applicability and usability of this kind of tools by expert curators in their daily tasks:

- Architecture: flexible and ready-to-scale architecture to support new features and integrate new services;
- Features: support for standard formats, integration with existing major services for document retrieval, integration with automatic annotation services, integration with existing state-of-the-art resources, flexible configuration of the annotation task and real-time collaboration functionalities;
- Usability: easy-to-understand interfaces with in-line annotations and interactions, and simple installation and configuration steps;
- Performance: fast document processing and representation.

In this article, we present Egas, a web-based platform for interactive biomedical information curation that intends to address the aforementioned demands, delivering a highly flexible and easy-to-use solution. It supports manual and automatic annotation of concepts and relations, together with in-line document representation and interaction. *De facto* standard knowledge bases are indexed and integrated to facilitate normalization of concept names. Real-time collaboration features are also provided to enhance curator’s communication and contribute to more consistent results. Moreover, Egas integrates on-demand configuration of the annotation task, namely annotators, concepts, relations and general annotation guidelines. Overall, based on the provided features and inherent characteristics, we strongly believe that Egas is a state-of-the-art solution to perform a large variety of biocuration tasks, ready to support information generation and keep current databases properly updated, and for text miners, biocurators and computational biologists, to perform a large variety of biocuration tasks, such as generating annotated corpora, collecting information from scientific literature and filtering literature for further research.

## Functionality

Egas is a web-based platform for biomedical TM and collaborative curation. It allows users to annotate texts with occurrences of concepts and relations between these concepts. The annotation tool follows what we termed an ‘annotation-as-a-service’ paradigm. Thus, document collections, users, configurations, annotations, back-end data storage, as well as the tools for document processing and TM, are all managed centrally. This way, a curation team can use the service, configured according to their requisites, taking advantage of a centrally managed pipeline. Moreover, Egas was created and developed with a strong focus on usability and simplicity, applying clean and self-explanatory user interfaces and interactions. Overall, the main goal is to facilitate interactive information mining, making the tasks of data understanding and respective information extraction as simple as possible.

**Figure 1.**
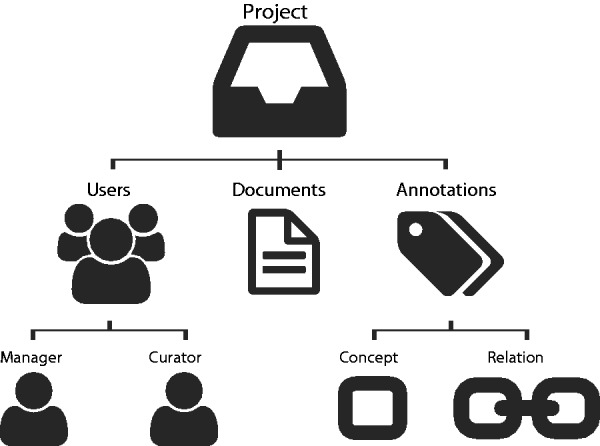
Egas organization based on projects, users, documents and annotations.

The tool is based on the idea of projects (Figure 1). A project consists of a curation task, performed by a team of curators on a collection of documents, and considering a predefined set of concept and relation types, as defined by the curation guidelines. The project manager is responsible for assigning users (curators) to the project for defining annotation guidelines, target concepts, relations and project accessibility (private or public). Thus, users can only annotate a document if they are associated to the respective project. Egas keeps track of all users operations regarding annotations, namely adding, changing and removing concepts and relations. It also automatically registers curation times of each user per document, providing such statistics for further analysis.

**Figure 2.**
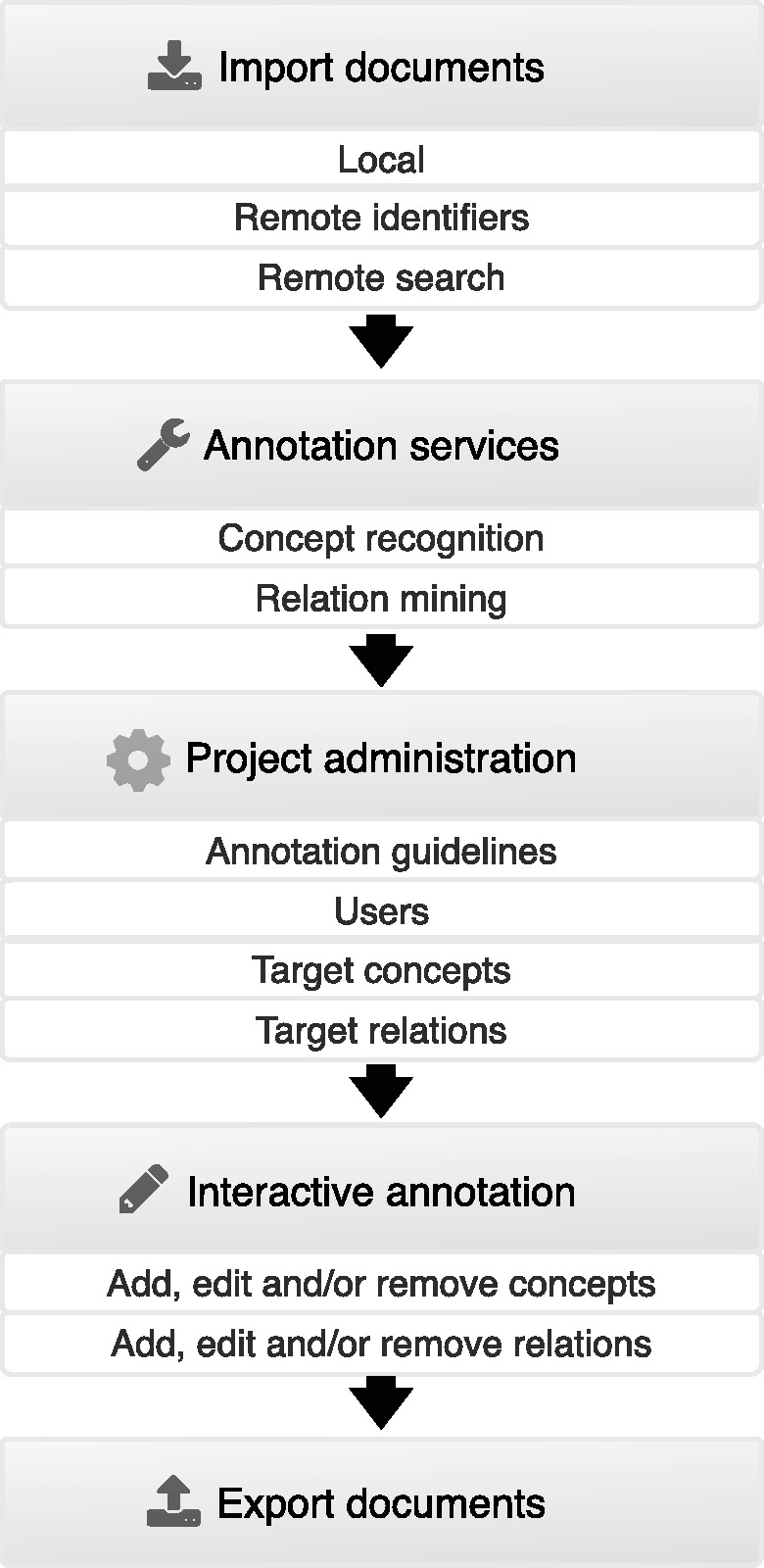
Typical usage pipeline of Egas.

Figure 2 summarizes the features provided by Egas and illustrates the typical usage pipeline. At first, to associate a collection of documents to a project, users can import documents from their devices in standard formats, containing raw or previously annotated texts, or use remote resources to import documents, either by providing a list of identifiers or by running remote searches on these resources. After importing documents to the project, they can be automatically annotated by using the available concept and relation annotation services. Afterwards, project administrators can freely define concept and relation types according to the requisites of the task. Additionally, each concept type can be associated to a knowledge base for normalization, and relations can be defined by specifying the types of the intervening concepts. Administrators can also upload documents describing the annotation guidelines and specify the users who are associated with the project. After this step, curators are able to annotate the available documents by adding, editing and removing concept and relation annotations, taking advantage of real-time collaboration features for faster and easier communication. In the end of the annotation process, users are able to export annotated documents and respective concept and relation annotations to standard formats.

### User interface

**Figure 3.**
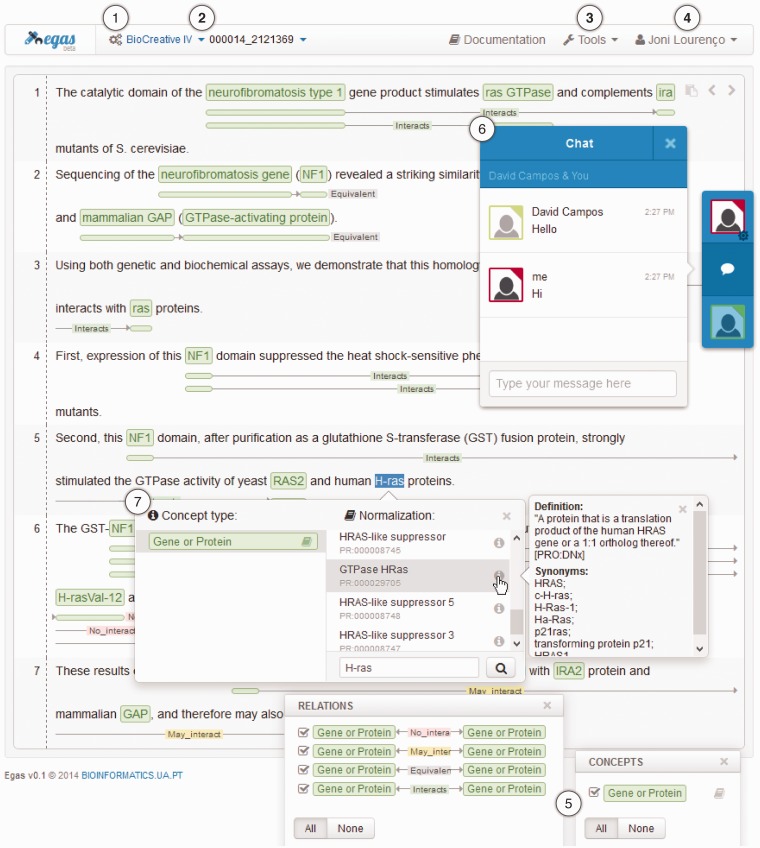
Egas main interface presenting a PubMed abstract (PMID 2121369) with annotated concepts and relations and emphasizing relevant interaction components/features: (1) project management; (2) project and document navigators; (3) processing tools; (4) account management; (5) concept and relation type visualization filters; (6) real-time collaboration; and, (7) concept annotation with normalization.

Egas was designed to be simple and easy-to-use, taking advantage of user-friendly interactions that are highly focused on the document annotation task. Figure 3 presents the Egas workspace, which contains six main action components for accessing the provided features:

1. Project management: manage and access project configurations, namely, users, concepts, relations, annotation guidelines and statistics;
2. Project and document navigators: navigate through different projects and documents;
3. Processing tools: access the integrated automatic annotation services, as well as the importing and exporting functionalities;
4. Account management: manage user account settings;
5. Concept and relation type visualization filters: select concept and/or relation types to be highlighted in the document viewer;
6. Real-time collaboration: communicate with other curators.

Concepts and relations are represented in-line, contributing to an improved annotation process by providing contextualized actions and rapid perception of the information added to the document. Concept annotations are highlighted with coloured boxes specific for each concept type, and to account for the complexity of the biomedical terminology, nested concept names are supported and carefully represented through overlayed boxes. On the other and, relations are displayed using directional lines below each sentence, tagged with the relation type and with boxes placed under the concepts that participate in the relation. The boxes have the same colour as the respective concept, making it easy to identify the entire relation. To simplify the analysis of the annotated concepts and relations, users can use the corresponding visualization filters to select the concepts and relations that are shown in the document viewer. By unchecking the checkbox associated with a specific concept or relation type, the corresponding coloured boxes are removed from the document viewer, cleaning the document representation and making its analysis more focused.

Finally, as part of the workspace, it is also possible to enable real-time collaboration features. That way, Egas provides instant feedback of user’s interactions within a document, such as adding, removing and/or changing concept and relation annotations. Thus, multiple users can change a document at the same time, showing exactly who changed what. A project chat is also available, which allows users to discuss details of the annotation task. Moreover, mouse-pointer-click position feedback is also provided, indicating where remote users clicked.

#### Concept and relation annotation

Information annotation is a key feature of Egas, which provides easy and interactive annotation of concepts and relations. Thus, to add a concept annotation, the user simply selects the chunk of text mentioning that concept, after which a menu is instantly shown allowing them to select the concept type and the concept identifier from a knowledge base, if required. Adding relations is just as straightforward, simply by clicking the two concepts while pressing the ‘Alt’ key and selecting the relation type in the pop-up menu. Right clicking an existing concept or relation allows removing that annotation or, in the case of relations, changing its type or direction.

#### Import and export documents

Import allows users to add documents to the currently selected project in three different ways. Local import allows users to select documents stored in their computer in three possible formats: raw text, A1 (http://brat.nlplab.org/standoff.html) and BioC (26). The two other options use remote servers to retrieve documents, either using lists of unique identifiers to select the documents or by searching remote literature indexing services. Currently, both PubMed and PubMed Central are supported, allowing to import abstracts and full-text documents, respectively. User queries are executed directly in the remote services, allowing logic operators such as ‘AND’ and ‘OR’, as well as MeSH type queries. After submitting the query, Egas presents a list of documents and allows the users to select the documents they want. On the other hand, export features are provided through a single interface, which allows users to select the documents to be exported and the output format. Egas currently supports two different formats: A1 and BioC.

#### Annotation services

The interface for calling automatic annotation services for specific documents was designed to be as flexible and adaptable as possible, to support services with different characteristics. Thus, Egas only requires the user to indicate the documents that should be annotated by the service. Afterwards, resulting annotations are loaded to Egas and presented in the document viewer.

#### Project management

Project management allows administrators to configure essential project characteristics, such as annotation guidelines, users, target concept and relation types, and access various statistics regarding the annotation process. The initial panel allows administrators to provide annotation guidelines for curators through in-line text and/or attached documents in standard formats, such as Adobe PDF and Microsoft Word documents. Moreover, users management allows inviting and removing users from each project by taking advantage of an e-mail–based invitation system. This panel also allows managing project administrators and pending issued invites. Besides the concept and relation types definition panels, Egas also provides a statistics panel, which allows administrators to collect detailed information regarding the annotation process per article and user, namely curation time and annotated concepts and relations. Exporting collected statistics for further analysis is also possible.

### Implementation

**Figure 4.**
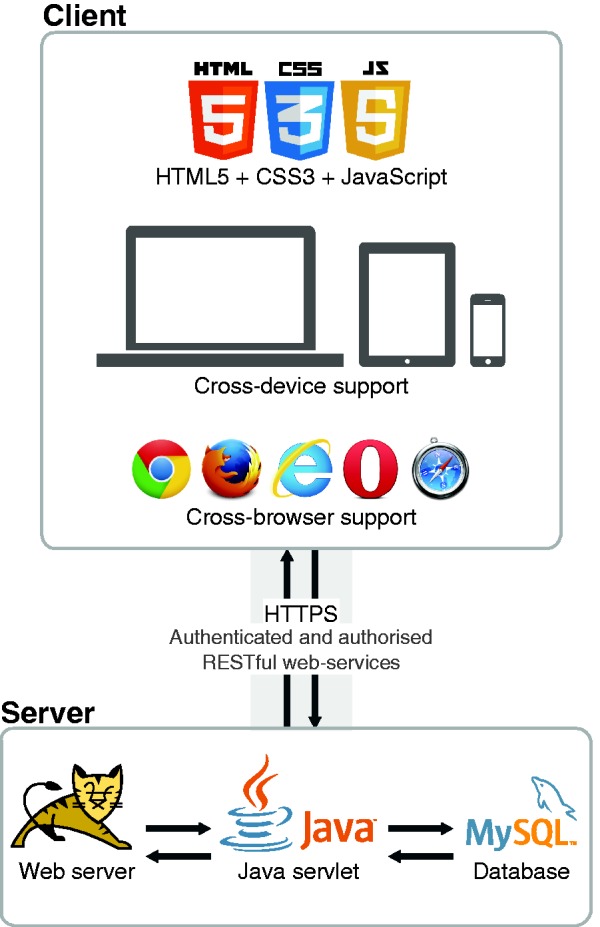
Egas architecture.

As a web-based platform, Egas intends to facilitate the access to an innovative and flexible solution for biomedical data curation, making it easily available for almost all internet-capable devices. Figure 4 illustrates the architecture of Egas, which is divided in two parts: client and server. The client-side is responsible for the direct interaction with users through their web-browsers, and the server-side is responsible for storing and processing all generated data. Both sides exchange data through a secured and encrypted channel using authenticated and authorized services.

The client-side was developed targeting compatibility and performance, through the application of standard web technologies, i.e. HyperText Markup Language, Cascading Style Sheets and JavaScript, which are supported by most commonly used web browsers on both desktop and mobile devices. The application of such web standard technologies also delivers fast representation of information. Thus, together with simple and fast client-side algorithms, we enable loading and presenting full-text documents with thousands of annotations in just a few seconds. For instance, considering one of the largest documents of the CRAFT corpus, which contains 3461 concept annotations, Egas spent only 3 s to present the document with respective annotations. On the other hand, a similar Scalable Vector Graphics (SVG) solution with in-line annotations required 14 s to load the same exact document. Thus, our approach presents an improvement of >4.5 times in terms of document representation speed, which provides a smooth and sophisticated navigation and interaction with the system.

The server side is responsible for storing all information in a unique resource, as well as providing the services to interact with that same data. All projects and respective users, documents, annotations and configurations are stored in a MySQL (http://www.mysql.com) relational database. Every processing task is available as a Representational State Transfer (REST) web service, enabling easy and fast integration in any development platform, such as web, desktop and mobile. Moreover, those web services are secured by requiring specific authentication and authorization per user. Additionally, to guarantee complete protection of exchanged data, the communication between client and server sides is performed through a secured and encrypted channel using Hypertext Transfer Protocol Secure (HTTPS).

#### Import and export documents

As previously described, Egas supports importing documents and respective annotations (when available) from local and remote servers, as well as export features to locally store curated documents. Three formats are supported for import and export: A1, BioC and raw text. A1 converter was developed in-house, and BioC support takes advantage of the publicly available BioC Java library (http://www.ncbi.nlm.nih.gov/CBBresearch/Dogan/BioC). The integration with remote servers is performed using web services, which already support retrieving specific documents by unique identifiers, or by submitting a search query. PubMed was integrated through the E-utility Simple Object Access Protocol (SOAP) web service (27), and PubMed Central using the Open Access (OA) REST web services (http://www.ncbi.nlm.nih.gov/pmc/tools/oa-service).

#### Annotation services

Automatic annotation services allow performing identification of specific concepts and/or relations in a custom set of documents using state-of-the-art algorithms. That way, users can call an automatic annotation service and posteriorly manually correct the provided annotations and/or add missing ones. Such approach intends to considerably decrease the amount of time spent in the manual curation process. Egas supports automatic annotation services through a unique and simple REST web-services interface. To comply with this, web services have to accept text as input and provide annotations following the A1 or BioC format as output. That way, it is straightforward to add new services to identify different concepts and/or relations. Moreover, Egas automatically adds concept and relation types provided by the service if they were not previously specified in the project configuration. Two different automatic annotation services for biomedical concept recognition and PPI mining are currently provided.

The concept identification service takes advantage of the BeCAS REST API (28) to provide annotations of genes and proteins, species, anatomical concepts, miRNAs, enzymes, chemicals, drugs, diseases, metabolic pathways, cellular components, biological processes and molecular functions. It was tested (5) on the CRAFT (29), AnEM (30) and NCBI disease (31) corpora, achieving F-measure results for overlap matching of 76% for genes and proteins, 95% for species, 65% for chemicals, 83% for cellular components, 92% for cells, 63% for molecular functions and biological processes, 83% for anatomical entities and 85% for diseases.

Regarding PPI extraction, as a state-of-the-art tool with fast processing times for real-time usage was not available as a service, we created a simple solution to provide relations between proteins and also indicate the possible presence of such relations, to support the manual annotation process. Thus, our PPI’s service does not only provide relations between proteins but also indicates the possible presence of such relations, supporting the manual annotation process. Thus, the following annotations are provided by this service: (i) protein concepts, (ii) relations between proteins, (iii) relations marking equivalent protein mentions (e.g. acronyms and long forms) and (iv) trigger words that may indicate the presence of PPIs. The service was implemented on top of Neji (5), using Gimli (32) to perform machine learning-based protein name recognition. BioThesaurus is used to normalize recognized names, through the application of prioritized dictionary matching, as described in (5). Equivalent protein relations are added using a simple abbreviation resolution technique, and PPIs are recognized through a rule-based approach using dependency-parsing trees. To do this, we first filter sentences by accepting only the ones that follow specific patterns, which have high probability of indicating PPIs:

- TRIGGER.*(of|between).*PRGE.*
-    (by|to|through|with|on|and).*PRGE
- TRIGGER.*containing.*PRGE.*and.*PRGE
- PRGE.*TRIGGER.*PRGE
- PRGE.*PRGE.*TRIGGER
- TRIGGER.*TRIGGER.*between. *PRGE. *and. *PRGE
- PRGE.*TRIGGER.*TRIGGER.*with.*PRGE
- PRGE.*PRGE.*TRIGGER.*PRGE

Afterwards, considering the previously collected trigger words as reference, a relation is considered if there is a directional path between the trigger word and two proteins, allowing a maximum of four hops.

#### Normalization

To offer normalization features in the easiest and fastest way as possible for biocurators, we indexed and integrated a rich set of biomedical knowledge bases. Apache Solr (http://lucene.apache.org/solr) was used to index the identifier, preferred name, synonyms and definition (if available) of each concept in these resources. For added flexibility and robustness, a separate index is used for each knowledge base. Additionally, as knowledge bases are available in heterogeneous formats, we developed scripts to automatically index ontologies in open biomedical ontologies (OBO) and web ontology language (OWL) formats and databases in structured query language (SQL) format. Resources available in custom formats require the development of custom-parsing algorithms. To cover the wide spectrum of biomedical knowledge, we decided to collect ontologies provided by OBO Foundry (33). Thus, a total of 110 ontologies were indexed, including NCI thesaurus (34), NCBI taxonomy (35), Protein Ontology (36), Gene Ontology (37), ChEBI (38) and Disease Ontology (39). Overall, more than 2 million entries are indexed and available for biocurators.

#### Real-time collaboration

Real-time collaboration features were implemented by taking advantage of TogetherJS (https://togetherjs.com) from Mozilla, a JavaScript library built on top of Node.js (http://nodejs.org) that simplifies the development of collaboration features. That way, all active users working in a document can observe the actions of adding, changing and removing concept and relations performed by other users. Additionally, every project has a dedicated chat, allowing users who are annotating different documents to discuss annotation guidelines, to minimize mistakes as much as possible.

## Results

### Experiment

Egas was tested in terms of applicability and user satisfaction in the BioCreative IV interactive annotation task (40), which intended to promote the development of useful TM solutions to fill the gap between the biomedical TM and biocuration communities, exploring the user-system interactions and hidden requirements. In that way, the task targeted the development of solutions to support interactive mining and/or triage of scientific documents.

The task organizers, together with a group of expert curators, defined a prioritized list of requirements that they considered more important to be available in such systems. The five more important system requirements were (i) highlighting of entities and relationships; (ii) processing of full texts; (iii) allowing manual mode for annotation; (iv) ability to edit results; and (v) ability to export curated results in standard formats. Each participating team developed and submitted its own approach to deal with the provided specifications. Moreover, each team had to propose a biocuration task to apply and test drive the presented system. Our proposal consisted of the identification and extraction of biomolecular events described over PubMed abstracts related to neuropathological disorders, including PPI, protein expression and post-translational modifications. To create the corpus for this task, a collection consisting of >135 000 PubMed abstracts was first obtained with the following query:

“Neurodegenerative Diseases”[MeSH Terms] OR “Heredodegenerative Disorders, Nervous System” [MeSH Terms] AND hasabstract[text] AND English[lang].

The documents were then ranked according to their relevance for extracting PPIs, using a SVM classifier (41) trained on the BioCreative III PPI Article Classification Task data (14). Such approach achieved an F-measure of 62% and an accuracy of 88%, when tested on the test part of the data. Finally, the top-ranked 100 documents were selected for the task.

Four curators were selected, and each was assigned 50 documents from the corpus to curate. Curators were asked to annotate 25 of their assigned documents using the available PPI annotation service described above, and the remaining 25 documents without using this service, to assess its impact on curation effort. In the first case, curators had to revise the automatically generated annotations, correcting any erroneous concept or relation annotations and adding missing ones. In the second case, curators had to annotate all mentions of protein names and all protein interactions described in each document. The tool recorded the time taken by each curator to curate each document, as well as the number of annotated concepts and relations.

## Results

Nine systems participated in the BioCreative IV IAT, targeting heterogeneous domains of application and differing significantly in the followed approaches, in terms of design, implementation and usability. Overall, four systems provided integrated triage features, eight systems supported concept recognition (five of those with normalization) and six systems enabled relation/event mining.

To properly evaluate the behaviour of the various systems, the BioCreative IV IAT organization committee built a detailed survey to subjectively rank and compare the different tools. Such survey covers various aspects of curators’ satisfaction, such as (i) overall reaction; (ii) comparison with similar systems; (iii) ability to complete tasks; (iv) design; (v) learning to use the application; and (vi) usability. The answers to each of the 23 questions were scaled from 1 (very bad) to 5 (very good). The obtained evaluation results were averaged and grouped in three categories: recommendation, rating and experience. Egas presented satisfying results in the three categories from the four curators, obtaining an average of 4.5 points in recommendation and 4.75 points in rating and experience.

Regarding the impact of automatic TM services, the application of these annotation algorithms significantly contributed to reduced curation times: for three of the four curators, the curation times were reduced by 1.5–4 times. However, we also observed that automatic services may contribute to biased annotations, as curators tend to be influenced by automatic annotations, accepting or performing slight changes without thorough analysis and reflection. Thus, automatic tools should follow the same standards and assumptions as defined by the annotation guidelines, a fact that must be carefully considered in any annotation task. For instance, if the automatic tool provides species names as part of protein names, and the annotation guidelines indicate otherwise, the final corpus can be easily inconsistent and with serious annotation mistakes, seriously degrading the final inter annotator agreement (IAA).

## Discussion

We believe that Egas presents various advantages for biocurators, in terms of usability and simplicity. These advantages are an added value for the biomedical community, contributing to a faster and more accurate annotation of biomedical information from scientific literature. Thus, we discuss the contributions of delivering a platform-as-a-service solution and of integrating real-time collaboration features.

### Biocuration-as-a-service

Following an application service solution, Egas enables on-demand creation and configuration of annotation projects, allowing supervisors to independently define target concepts and relations, invite curators and define annotation guidelines. Moreover, during the annotation process, supervisors can change any of the settings on-demand, obviously respecting consistency requirements. For instance, a user cannot delete a concept type if a relation type is using it. Additionally, the statistics dashboard allows administrators to actively supervise the performed work, providing valuable information regarding curation time and the amount of concepts and relations per article and user. Such active management and supervision is only possible by taking advantage of the integrated annotation task management features, which we believe is an added value for biocurators.

Egas also facilitates concept normalization by integrating and indexing a complete set of knowledge bases, offering heterogenous information targeting different domains of interest. That way, the presented platform positively responds to the needs of the most different curation tasks. The integration of such resources considerably facilitates biocurators tasks, as they do not have to acquire a deep understanding of knowledge bases and/or develop any kind of scripts to process and integrate them. Thus, users can take advantage of such ontologies by simply associating a concept type with a specific normalization resource.

Finally, Egas also integrates annotation services to provide automatic identification of concepts and relations. As previously discussed, such integration may contribute to improved curation speeds, resulting in more time available to annotate more documents. As the interaction with different automatic annotation services is performed through a single and self-explanatory interface, biocurators do not need any kind of expertise to take advantage of such advanced technologies. Overall, this simple integration of annotation services allows biocurators to easily take advantage of high-end and advanced biomedical TM solutions, an approach that may streamline the communication and collaboration between TM and biocuration communities.

By delivering a platform-as-a-service, Egas significantly facilitates the set-up and on-demand configuration of annotation tasks. Additionally, as many curation tasks may work with sensitive data, we considered security as one of the most important characteristics of our system. That way, all communications between clients and the server are performed through secured channels, using HTTPS. Moreover, all actions that interact with the centralized database are carefully authorized and authenticated, considering the user permissions.

### Real-time collaboration

In a classic annotation process, those responsible for the task start by specifying a target domain and by defining the annotation guidelines, where they describe target concepts, relations and present examples of what to annotate. Afterwards, each curator has access to the annotation guidelines, interprets them and starts annotating the set of documents that he/she was assigned to. During this process, frequent discussions among annotators to resolve and document ambiguous cases and repeated verification of the annotated data against the guidelines are performed, to ensure annotation quality. In the end, IAA may be calculated to obtain a feedback regarding generated information consistency among curators. However, some research works (42) focused their efforts on annotating more documents with high quality, guaranteed by active supervision and correction of mistakes, rather than annotating repeated documents to obtain IAA scores. Based on this, we strongly believe that the definition of annotation guidelines and the active discussion and iterative correction of annotations and respective guidelines is one of the most important aspects of the annotation process. Thus, through Egas, annotation task supervisors around the globe can work together to define the first version of annotation guidelines, taking advantage of the real-time feedback of concept and relation annotations, and of the chat to discuss mistakes and ideas. Additionally, as annotation guidelines are integrated in the platform, all supervisors can contribute to their improvement, and all participants have access to the most updated version. After starting the annotation process, curators can use real-time collaboration features to discuss with each other the interpretation of annotation guidelines using the chat, and supervisors can observe curators’ work, correcting and discussing mistakes, and possibly improving the guidelines. In conclusion, through real-time features, we intend to promote the active involvement of both supervisors and curators in the annotation process, to deliver improved information consistency and quality.

## Conclusion

This article presents Egas, a complete platform for scientific literature curation, focused on usability, simplicity, security and integration. It offers highly usable interfaces for manual and automatic in-line annotation of concepts and relations. A comprehensive set of knowledge bases are integrated and indexed to provide straightforward concept normalization features. Moreover, real-time collaboration and conversation functionalities allow discussing details of the annotation task as well as providing instant feedback of curator’s interactions. Egas also provides interfaces for on-demand management of the annotation task settings and guidelines, and supports standard formats and literature services to import and export documents. With Egas, we participated in the BioCreative IV interactive annotation task, targeting the assisted identification of PPIs described in PubMed abstracts related to neuropathological disorders. When evaluated by expert curators, it presented good results regarding usability, reliability and performance. The application of automatic annotation services presented considerably reduced curation times. Moreover, Egas showed superior document processing and representation speeds, which is a significant added value and contribution to a smoother annotation process. Overall, Egas presents various advantages for the biomedical community, streamlining the collaboration between supervisors and curators, and simplifying the set-up and on-demand configuration of the annotation task, using integrated knowledge bases and automatic annotation services. These contributions, together with the presented results, show that Egas is a state-of-the-art solution to perform a large variety of biocuration tasks, ready to grow and to be integrated with any major platform to support information generation and keep current databases properly updated in a consistent way.

### Future work

Egas provides a rich set of features that make it an innovative solution with many advantages for the biocuration community. However, we believe this is the baseline of an advanced platform to support interactive mining of biomedical information. Thus, there are many features that can be integrated in Egas to further improve it, delivering enhanced assistance to biocurators. At first, we plan to support more knowledge bases for normalization (e.g. ontologies from BioPortal, Uniprot and UMLS), more input and output formats (e.g. PDF and SQL) and more automatic annotation services (e.g. DDI and events), including confidence values of predicted annotations. Regarding annotation, we intend to support event extraction through a unique and easier-to-understand in-line representation and provide features to add notes and text passages as supporting information for concept, relation and/or event annotations. Document triage features may be also integrated, through the development of a master index and respective services with automatically annotated concepts, relations and scores of multiple document ranking strategies. To simplify document management, we also intend to support document comparison and provide features to search for specific terms in the set of documents in the project. Finally, to promote wider usage, we intend to create standalone server and respective configuration scripts for simplified distribution and installation in local machines.
